# Thermodynamics and kinetics of the F_o_F_1_-ATPase: application of the probability isotherm

**DOI:** 10.1098/rsos.150379

**Published:** 2016-02-10

**Authors:** Brian Chapman, Denis Loiselle

**Affiliations:** 1 Honorary Principal Research Fellow, School of Applied and Biomedical Science, Faculty of Science and Technology, Federation University Australia, Victoria, Australia; 2 Department of Physiology and the Auckland Bioengineering Institute, The University of Auckland, Auckland, New Zealand

**Keywords:** thermodynamics, kinetics, chemiosmosis, stoichiometry, thermodynamic efficiency

## Abstract

We use the results of recent publications as vehicles with which to discuss the thermodynamics of the proton-driven mitochondrial F_*o*_F_1_-ATP synthase, focusing particularly on the possibility that there may be dissociation between rotatory steps and ATP synthesis/hydrolysis. Such stoichiometric ‘slippage’ has been invoked in the literature to explain observed non-ideal behaviour. Numerical solution of the Rate Isotherm (the kinetic equivalent of the more fundamental Probability Isotherm) suggests that such ‘slippage’ is an unlikely explanation; instead, we suggest that the experimental results may be more consistent with damage to the enzyme caused by its isolation from the biomembrane and its experimental fixation, resulting in non-physiological friction within the enzyme's rotary mechanism. We emphasize the unavoidable constraint of the Second Law as instantiated by the obligatory dissipation of Gibbs Free Energy if the synthase is to operate at anything other than thermodynamic equilibrium. We use further numerical solution of the Rate Isotherm to demonstrate that there is no necessary association of low thermodynamic efficiency with high metabolic rates in a bio-world in which the dominating mechanism of metabolic control is multifactorial enzyme activation.

## 1. Introduction

In 1978, Peter Mitchell [1] was awarded the Nobel Prize for Chemistry for his discovery of the chemiosmotic mechanism of ATP synthesis, in which the mitochondrial electron transport system establishes a separation of protons across the inner mitochondrial membrane. The resulting electrochemical proton motive force (PMF) provides the energy for the synthesis of ATP from its breakdown products. Three years earlier, Paul Boyer [2] had postulated that oxidative phosphorylation might be driven by sequential conformational changes in a mitochondrial membrane-bound enzyme. Two decades later, John Walker's group [3] deduced the three-dimensional crystalline structure of the bovine mitochondrial F_1_-ATPase at 2.8 Å, sufficient precision to declare that ‘interconversion of … states may be achieved by rotation of the *α*_3_*β*_3_ subassembly relative to an *α*-helical domain of the *γ*-subunit’ [3], p. 621. For these respective theoretical and experimental achievements, Boyer and Walker shared the 1997 Nobel Prize for Chemistry. In the same year, a stunningly conceived and executed experiment by Noji *et al.* [4], involving attachment of a single, fluorescently tagged, 2.6 μm actin filament to the *γ*-subunit, provided direct visual proof that the bacterial F_1_-ATPase indeed operates with a rotary mechanism—its central *γ*-shaft spinning in a stationary stator barrel comprising three *α* and three *β* subunits. Publication of that article generated fascinating comment in *Nature* [5].

Since publication of the seminal paper by Noji *et al.*, numerous variations on its theme have been reported with a view: (i) to corroborating its results [6]; (ii) to characterizing its temperature-sensitivity [7]; (iii) to achieving increased speed of rotation by using 80 nm [8] or 40 nm [9] gold beads, or magnetic tweezers and magnetic beads, thereby revealing sawtooth steps at 120° rotations [10], culminating in the capture of sub-sawtooth accelerations and decelerations within individual sawtooth repeats of a single power stroke [11]; or (iv) to improving visualization of nucleotide binding by capitalising on the fluorescence responses of Trp mutants [12]. In parallel, a number of mathematical models have been developed to mimic experimental observations of: (i) steady-state kinetics [13,14], (ii) rotor dwell times [12,15], (iii) torque-dependence of rotational velocity [16], (iv) the role of electric fields localized at the entry and exit ports of protons to drive rotation of the central c-ring [17], (v) the maximal thermodynamic efficiency of single F_1_-ATPase molecules [18,19], (vi) the variation of efficiency with the stoichiometry of the c-subunit [20] and, most recently, (vii) a formal methodology for relating rate constants and free energies to the stalling angle of the rotor [21].

In the following, we focus on the thermodynamics of the F_*o*_-F_1_-ATPase in both its forward (i.e. the synthesis of ATP from ADP and Pi) and reverse (i.e. hydrolysis of ATP to ADP and Pi) directions. Our principal focus is on the publication by Toyabe *et al*. [22] (hereinafter referenced as ‘the Authors’), which we use as a vehicle for commenting on thermodynamic reversibility and related issues. These authors reported a linear relationship between the logarithmic ratio of forward to reverse 120° steps in the rotation of the *γ*-shafts of single molecules of F_1_-ATPase and the magnitude of the torque applied by electrorotation methods via a molecular probe. Thus,

1.1 $$k_{B}T\ln\frac{p_{s}}{p_{h}}=(N-N_{\mathrm{stall}})d,$$

where *k*_*B*_ is the Boltzmann constant, *T* is the absolute temperature, *p*_*s*_ and *p*_*h*_ are the fractions of rotational steps observed in the synthetic and hydrolytic directions of the enzyme, respectively, under applied torque *N*, *p*_*h*_=(1−*p*_*s*_), and 120° is the amount of shaft rotation associated with the hydrolysis or synthesis of one molecule of ATP. The experimental data determined values for the two fitting parameters in the linear relationship as *N*_*stall*_=31.2 pN nm rad^−1^ and *d*=55°.

In discussing their results, the Authors noted the coincidence between the experimentally controlled free energy of ATP hydrolysis/synthesis (Δ*μ*=65.2 pN nm) and the work done by rotating the *γ*-shaft through 120° under the stalling torque at which *p*_*s*_=*p*_*h*_, *W*_*stall*_=*N*_*stall*_×120°=65.3 pN nm. This finding moved them to state that

… [the] F_1_-motor serves as a highly efficient mechanochemical free-energy transducer of F_*o*_F_1_-ATP synthase at almost 100% thermodynamic efficiency. For such a high efficiency to be achieved, a tight mechanochemical coupling is expected, that is, one ATP is hydrolysed during every hydrolytic-direction step, whereas one ATP is synthesized during every synthetic direction step.

[22], p. 17 954

However, becoming troubled by the mismatch between the known chemically fruitful stepsize (*d*=120°) and their experimentally fitted value of *d*=55°, the Authors went on to speculate that their observations may have been contaminated with chemically fruitless jumps (‘trial steps’). It is this interpretation that prompts us to respond.

Our response has three objectives: firstly, to examine by numerical solution of the Rate Isotherm—the kinetic equivalent of the more fundamental Probability Isotherm (see equation (2.2) below)—the possible influence of chemically fruitless jumps as postulated by Toyabe *et al.* [22]; secondly, to offer an alternative explanation for the mismatch between the known stepsize of rotation of the *γ*-shaft of a single F_1_-ATPase molecule and the Authors’ experimentally derived value; and thirdly, to disentangle the common confusion between the concepts of tight stoichiometric coupling and high thermodynamic efficiency.

## 2. Ideal behaviour

Equation (1.1) expresses the molecular free energy dissipation, Δ*μ*_*diss*_, associated with net stepwise rotation of the *γ*-shaft through 120° in terms of the probability ratio of forward to backward stepping, thus

2.1 $$\Delta \mu_{\mathrm{diss}}=k_{B}T\ln\frac{p_{s}}{p_{h}}.$$

It is a particular case, at the molecular level, of the Probability Isotherm coined^1^ by Chapman *et al.* [23], which expresses the molar free energy dissipation, Δ*G*_*diss*_, of any reaction, however complex, in terms of the probability ratio for the overall forward and backward processes, thus

2.2 $$\Delta G_{\mathrm{diss}}=RT\ln\frac{p_{f}}{p_{b}},$$

where *R* is the ideal gas constant (the molar equivalent of the Boltzmann constant).

**Figure 1.**
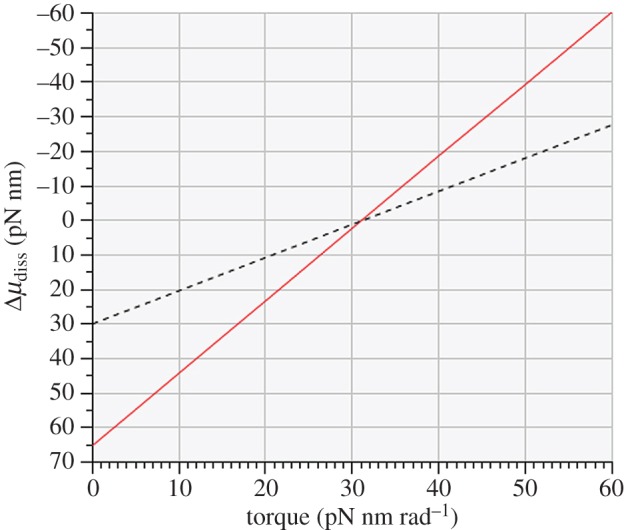
Linear relationship between molecular free energy dissipation and applied torque for idealized 120° rotational steps by the *γ*-shaft of the F_1_-ATPase (solid line). The ordinate intercept at zero torque is equal to − Δ*μ*_*ATP*_ while the equilibrium or stalling condition occurs at an applied torque of 31.2 pN nm rad^−1^. The dashed line shows the experimental result derived by Toyabe *et al.* [22].

It is apparent from the experimental design used by the Authors that, in the absence of any applied torque, the molecular free energy dissipated by the F_1_-ATPase for each 120° step would be exactly equal to Δ*μ*_*ATP*_, the molecular free energy available from ATP hydrolysis under their experimental conditions, given as 65.2 pN nm. Conversely, the ‘stalling’ or equilibrium condition for this stepping process would determine that Δ*μ*_*diss*_=0 and that *p*_*s*_=*p*_*h*_. The ideal linear relationship between the logarithmic probability ratio and the molecular free energy dissipation given by equation (2.1) is shown in figure 1 (solid line) along with the experimentally derived relationship (dashed line) derived by the Authors.

## 3. Explaining non-ideal behaviour

The Authors stated that the departure from ideal behaviour reflected in the dashed line of figure 1 ‘provides valuable information about the mechanochemical coupling’ [22], p. 17 954. In discussing this issue they invoked ‘rotational steps without the completion of chemical cycles’ which could be either ‘on-pathway’ (rotations that don’t run as far as being completed by chemical change) or ‘off-pathway’ (rotations that run to completion but with no associated chemical change—i.e. stoichiometric ‘slippage’). Furthermore, the Authors implicitly allowed that their observations may have been contaminated by these chemically fruitless rotations, that they had no means of distinguishing them from the tightly coupled chemically completed rotations, and that such fruitless rotations occurred with a probability ratio of unity for the two directions independently of the applied torque. Thus, they suggested that the true occurrence frequencies of the synthetic and hydrolytic steps, *n*_s_ and *n*_h_, respectively, would require to have added to them an unknown frequency of chemically futile steps, *m*, which would make the logarithm of their observed ratio, *p*_s_/*p*_h_, less in magnitude than that of the logarithm of the ratio of the true tightly coupled steps, *n*_s_/*n*_h_ and so would reduce the slope of the linear relationship plotted in figure 1. However, this line of explanation is fraught with difficulty as we now show by constructing some numerical examples that incorporate chemically futile cycles.

**Figure 2.**
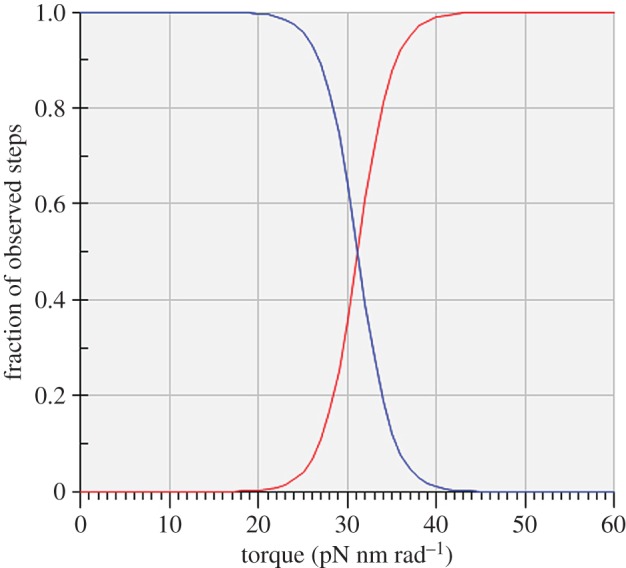
The fraction of steps in the synthetic direction, *p*_s_ (red), and in the hydrolytic direction, *p*_h_=1−*p*_s_ (blue), under applied torque for idealized 120° rotational steps by the *γ*-shaft of the F_1_-ATPase. The intersection of the two probability functions at the equilibrium or stalling condition occurs at an applied torque of 31.2 pN nm rad^−1^.

Because the Authors did not specify the absolute unidirectional rates corresponding to their unidirectional probabilities as plotted in their fig. 6*a*, we have made some assumptions based on information about absolute rates given in relation to their figs. 2 and 3. We begin by assuming that the maximum molecular turnover rate of the 120° step for the F_1_-ATPase in the absence of applied torque is 35 s^−1^, based on a maximum observed rotation rate just short of 12 Hz (their fig. 3). The resulting uncontaminated (idealized) unidirectional occurrence frequencies are shown in figure 2.

**Figure 3.**
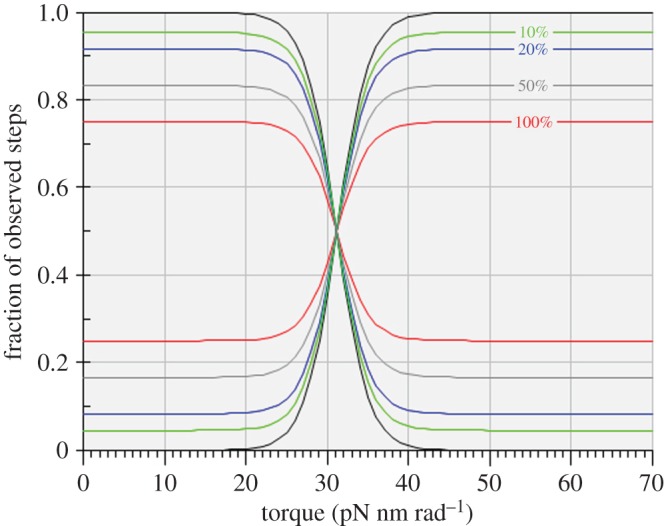
The effect of ‘contamination’ by chemically fruitless steps on the fraction of observed steps in the synthetic direction (from lower left to upper right), and in the hydrolytic direction (from upper left to lower right), under applied torque for idealized 120° rotational steps by the *γ*-shaft of the F_1_-ATPase. The intersection of each pair of functions at the equilibrium or stalling condition occurs at an applied torque of 31.2 pN nm rad^−1^. The levels of ‘contamination’ are zero (black, unmarked), 10% (green), 20% (blue), 50% (grey) and 100% (red).

## 4. Including ‘contamination’ with chemically fruitless rotational steps

**Figure 4.**
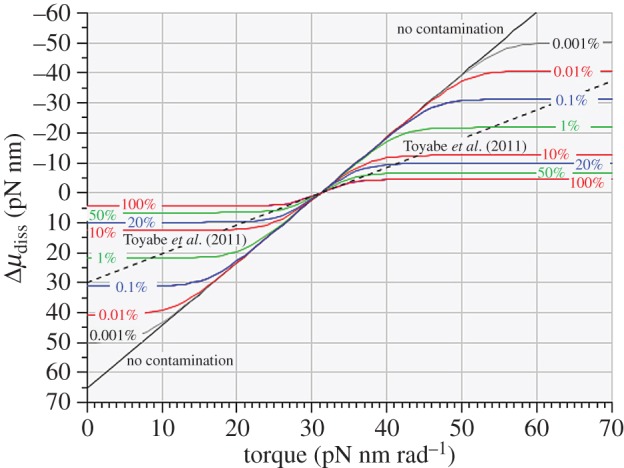
Relationship between molecular free energy dissipation and applied torque for idealized 120° rotational steps by the *γ*-shaft of the F_1_-ATPase with different levels of ‘contamination’ by chemically fruitless rotational steps ranging from ‘no contamination’ to 100% ‘contamination’ as described in the text. The uncontaminated ordinate intercept at zero torque is equal to − Δ*μ*_*ATP*_ while the equilibrium or stalling condition occurs at an applied torque of 31.2 pN nm rad^−1^. The graph also reproduces the results obtained by Toyabe *et al.* [22]—black dashed line.

We now equate the maximum unidirectional rate of chemically fruitful rotational steps of 120° to 35 s^−1^ and make a working assumption that the stall condition determines equal and opposite unidirectional rates of 17.5 s^−1^. This working assumption is a gross simplification relative to what the Authors’ original traces indicate in their fig. 2, where the maximum unidirectional stepping rates are dependent on torque, and where the ‘stalled’ rate of stepping is markedly slower than any of the non-stalled stepping rates; however, this simple working assumption allows us to learn from the predicted effects of levels of supposed ‘contamination’ that very likely exceed any levels supposed by the Authors. We have included different levels of ‘contamination’ by setting them equal to different percentages of the unidirectional ‘stalled’ rate of 17.5 s^−1^ ranging from 0.001% to 100%, applied equally to both directions of rotation. The effects of such ‘contamination’ on the relationship between ‘observed’ unidirectional stepping rates and torque are shown in figure 3 and the effects on the relationship between ‘observed’ free energy dissipation and torque are shown in figure 4.

Two key observations are evident from these calculations. Firstly, as shown in figure 3, such contamination as that postulated by the Authors results in asymptotic unidirectional probabilities that fall well short of unity, quite unlike the unidirectional probability distributions plotted in their fig. 6*a* which are indistinguishable from distributions containing no ‘contamination’. Secondly, as shown in figure 4, all levels of ‘contamination’—whether in trace amounts or approaching parity with true chemomechanically coupled steps—convert the uncontaminated rectilinear relationship between the logarithmic probability ratio and torque to a sigmoid relationship that is not compatible with the rectilinear experimental plot reported by the Authors.^2^

We conclude, therefore, that ‘contamination’ with chemically fruitless rotatory steps is not a satisfactory explanation for the difference between the Authors’ experimental result of their fig. 3*b* (represented by the dashed line in our figures 1 and 4) and the theoretical relationship determined by the Probability Isotherm as plotted in our figure 1 (solid red line) and figure 4 (labelled ‘no contamination’).

## 5. An alternative explanation

We consider there to be no need to invoke supposed chemically fruitless rotational steps to explain the non-ideal behaviour reported by the Authors. Instead, we suggest that the discrepancy between ideal behaviour determined by the Probability Isotherm and that reported by the Authors may be reconciled by postulating molecular friction between the rotor and its housing in the F_1_-ATPase molecule, the work of overcoming such friction in each 120° rotatory step being quantized, independent of stepping rate, independent of stepping direction and equal to approximately half of Δ*μ*_ATP_. This would have the reported effect of roughly halving the slope of the linear relationship between the logarithmic probability ratio and the applied torque, along with halving the value of the ordinate intercept at zero applied torque.

If this is accepted as the explanation, then two likely corollaries emerge. Firstly, there is no evidence for any kind of stoichiometric slippage between rotation and ATP hydrolysis/synthesis. Secondly, it does not stretch credulity to suppose that such significant friction is a result of ‘damage’ done to the enzyme in the process of isolating it from its natural membrane-bound environment and fixing it artificially for the experimental set-up. Friction in such a preparation has also been averred for the isolated F_1_-ATPase in the ingenious experimental measurement of angular velocity of *γ*-shaft rotation by Martin *et al*. [11]; these authors also noted that the variable angular velocity militated against the attainment of 100% thermodynamic efficiency in such preparations. In support of this interpretation is the evidence from the Authors’ fig. 2 that the magnitude of the free energy dissipation gradient is far more deterministic of the absolute rate of rotational stepping than any thermally determined Brownian events. In this connection, it is worth emphasizing that any expression such as the Probability Isotherm, which contains either the ideal gas constant (*R*) in its molar form or the Boltzmann constant (*k*_*B*_) in its molecular form, is ultimately an idealization applicable to inert gases at low temperature and pressure. Such idealized systems know nothing of inelastic collisions or the concept of friction. A single molecule of F_*o*_F_1_-ATP synthase—especially when considered in the context of a ‘battery’ of identical macromolecules arranged anisotropically in a biomembrane—is very far removed from this idealized kinetic world.

Nonetheless, the thermodynamic requirement for free energy dissipation, as determined by the Probability Isotherm, is inescapable. In particular, note that our theoretical development (figure 4) explicitly disallows values of thermodynamic efficiency in excess of 100% or any supposed free energy dissipation in excess of the free energy available, as claimed by Kawaguchi *et al.* [16]. Therefore, we turn now to examining some idealized situations in which the Probability Isotherm can be applied to profitable illumination of some unexpected relationships between thermodynamic efficiency and reaction rates in a bio-world in which metabolic control mechanisms are primarily directed towards controlling the level of catalysis.

## 6. Mechanochemical coupling and thermodynamic efficiency

The concepts of coupling (whether chemiosmotic or chemomechanical), heat production and thermodynamic efficiency are often confused and confounded in the bioenergetics literature. A convenient starting point for this part of the discussion is to note four concepts to be applied to discussion of chemiosmotic mechanisms in which free energy transduction occurs between chemical change (ATP hydrolysis or synthesis) and electro-osmosis (change in transmembrane PMF).

(i) To a first approximation, the molar enthalpy of electro-osmotic change is negligible relative to that of ATP hydrolysis or synthesis.
(ii) It is misleading to speak of the F_*o*_F_1_-ATP synthase as having a rotary mechanism that ‘transduces energy between chemical free energy and mechanical work’ as suggested by the Authors. The *free* energy transduction is purely chemiosmotic, i.e. between the electro-osmotic *free* energy change (altered PMF) and the chemical *free* energy change (ATP synthesis or hydrolysis).
(iii) Free energy *dissipation* (i.e. NOT transduction) is what *must* happen if net chemiosmotic reaction is to occur; without the dissipation of free energy, nothing happens.
(iv) The highly ordered crystalline structure of the F_*o*_F_1_-ATPase suggests strongly that the safest assumption to be made about its chemiosmotic stoichiometry is that it is fixed, i.e. not variable or susceptible to ‘slippage’, regardless of whether ATP synthesis or hydrolysis is being considered. Without the truth of this assumption it is difficult to imagine how the experimental work under consideration could have led to the conclusion that the enzyme operates at close to 100% thermodynamic efficiency.

## 7. Chemiosmotic heat production

The first concept above allows us to make predictions about heat production by the chemiosmotic system defined by the F_*o*_F_1_-ATP synthase and its associated PMF.

Firstly, consider ATP synthesis driven by the PMF. Regardless of the thermodynamic efficiency at which the ATP synthase may function, the heat absorbed per mole of ATP synthesized will be almost entirely accounted for by the magnitude of the enthalpy of ATP synthesis. The more efficient the transduction, the less will be the heat generated by entropy *creation*^3^ within the system and its surroundings, but the less will be the heat absorbed from the surroundings by entropy *exchange* (the system gains entropy through the less ordered state associated with ‘spending’ the PMF). The inverse relationship between created and exchanged entropy as thermodynamic efficiency varies means that a relatively constant amount of heat will be absorbed per ATP molecule synthesized. At high thermodynamic efficiency, there is relatively less entropy creation (free energy dissipation) deriving, for example, from the internal viscosity of the rotary motor and the internal resistance of the F_*o*_ proton channel.

Conversely, consider PMF generation driven by ATP hydrolysis. Regardless of the thermodynamic efficiency, the heat lost per mole of ATP hydrolysed will be almost entirely accounted for by the magnitude of the enthalpy of ATP hydrolysis. The more efficient the transduction, the less will be the heat generated by entropy *creation*within the system and its surroundings, but the more will be the heat lost to the surroundings by entropy *exchange* (the system loses entropy through the more ordered state associated with generating the PMF). The inverse relationship between created and exchanged entropy as thermodynamic efficiency varies means that a relatively constant amount of heat will be generated per ATP molecule hydrolysed. At high thermodynamic efficiency, there is relatively less entropy creation (free energy dissipation).

It should be noted that these considerations remain true regardless of the value of the chemiosmotic reaction's actual H^+^/ATP stoichiometry, whether it be fixed or ‘slippery’.

## 8. Mechanical ‘work’ during chemiosmosis

It is a necessity of the Second Law that free energy must be dissipated if there is to be any net chemiosmotic reaction. Free energy dissipation as heat for the F_*o*_F_1_-ATPase (or ATP synthase) comprises the *internal work*of overcoming the internal viscosity and resistance of the rotary mechanism and its associated proton channel. In no sense whatsoever is there free energy *transduction* (conservation) as useful mechanical work under physiological conditions. It is only the extraordinarily ingenious experimental conditions devised by modern molecular biologists, especially including the Authors, that make it possible to add *external mechanical work* to the thermodynamic burden of the enzyme and so produce true chemomechanical free energy transduction; no such mechanical transduction (free energy conservation as mechanical work) occurs in the normal operation of this enzyme.

## 9. The thermodynamic necessity for free energy dissipation

The kinetic equivalent of the Probability Isotherm given by equation (2.2) is the corresponding ‘Rate Isotherm’ [23], thus:

9.1 $$\Delta G_{\mathrm{diss}}=RT\ln\frac{r_{f}}{r_{b}},$$

where *R* and *T* are as defined previously, and *r*_*f*_ and *r*_*b*_ are the respective forward and backward unidirectional rates. This kinetic expression of the Second Law is independent of mechanism and is applicable to all conceivable chemical and/or chemiosmotic reactions, however complex, under any conditions [26]. It is amenable to quantitative analysis under any experimental conditions in which it is possible to define the unidirectional rates.

In equation (9.1), the absolute values of *r*_f_ and *r*_b_ (but not their thermodynamically determined ratio) will depend on the level of enzyme catalysis. A reaction that is at thermodynamic equilibrium (Δ*G*_diss_=0) will have a net rate of zero and a unidirectional rate ratio of unity. A reaction proceeding at high thermodynamic efficiency (close to equilibrium) at a given net rate will be doing so with a small rate ratio (therefore, a small Δ*G*_diss_), while the same reaction proceeding at lower thermodynamic efficiency (further from equilibrium) at the same given net rate will be doing so with a larger rate ratio (therefore, a larger Δ*G*_diss_). So, if it is required that a given reaction is to proceed at a particular net rate, the thermodynamic efficiency at which the net rate will be achieved will depend on the level of catalysis: for a given net rate the higher the level of catalysis (enzyme activity), the smaller the rate ratio, the smaller the free energy dissipation, and so the higher the thermodynamic efficiency will be.

There is much to be learnt by studying some simple numerical examples of the implications of equation (9.1). We now do this by considering an ATPase/ATP synthase with a fixed stoichiometry of $3.\dot{3}\;H^{+}/\mathrm{ATP}$ [27] proceeding at a net rate, *r*_f_−*r*_b_, of ATP hydrolysis or synthesis equal to 10 mM s^−1^ at different levels of thermodynamic efficiency determined by different levels of catalysis which have been simulated by setting the rate ratio, *r*_f_/*r*_b_, equal to six different values increasing from 1.01 to 10^4^. The resulting instantaneous thermodynamic conditions are shown in table 1 for an assumed Δ*G*_ATP_ of −50 kJ mol^−1^ for hydrolysis at a temperature of 310 K, thereby determining an equilibrium PMF of 155.46 mV (mitochondrial matrix negative). The data in table 1 thus illustrate numerically the general points made above concerning the relationship between level of catalysis (a rough proxy for which is the magnitude of *r*_f_) and the thermodynamic efficiency at which the enzyme works to produce a given rate of net chemiosmotic reaction.

**Table 1.** Energetic consequences of catalytic activity determined according to the Rate Isotherm for a reaction proceeding at a fixed net rate of 10 mM s^−1^.

				ATPase		ATP synthase	
*r*_f_/*r*_b_	*r*_f_ (mM s^−1^)	*r*_b_ (mM s^−1^)	Δ*G*_diss_= $\mathrm{RT}\ln(r_{f}/r_{b})$ (kJ mol^−1^)	PMF^*a*^ (mV)	efficiency (%)	PMF^*b*^ (mV)	efficiency (%)
1.01	1010	1000	0.026	155.38	99.95	155.54	99.95
1.1	110	100	0.246	154.70	99.51	156.23	99.51
11	11	1	6.177	136.25	87.65	174.67	89.00
101	10.1	0.1	11.889	118.50	76.22	192.43	80.79
10^3^	10.01001	0.01001	17.795	100.13	64.41	210.79	73.75
10^4^	10.001	0.001	23.727	81.69	52.55	229.24	67.82

^*a*^The PMF in this column shows that generated from ATP hydrolysis (assuming a free energy gradient of Δ*G*_ATP_=−50 kJ mol^−1^) in the face of the given free energy dissipation, Δ*G*_diss_, determined according to the Rate Isotherm. It is calculated as PMF=(Δ*G*_ATP_+Δ*G*_diss_)/*nF*, where *F* is the Faraday constant and $n=3.\dot{3}\;H^{+}/\mathrm{ATP}.$ The corresponding thermodynamic efficiency is calculated as (Δ*G*_ATP_+Δ*G*_diss_)/Δ*G*_ATP_. ^*b*^The PMF in this column is that required to synthesize ATP against its free energy gradient of 50 kJ mol^−1^ in the face of the given free energy dissipation, Δ*G*_diss_, determined according to the Rate Isotherm. It is calculated as PMF=(Δ*G*_ATP_−Δ*G*_diss_)/*nF*. The corresponding thermodynamic efficiency is calculated as Δ*G*_ATP_/(*nF*×PMF) or Δ*G*_ATP_/(Δ*G*_ATP_−Δ*G*_diss_).

While the efficiency values in table 1 are larger for the ATP synthase than they are for the ATPase, this says nothing fundamental about the net direction in which the reaction is proceeding; it is merely a happenstance of the method of illustration used in each case whereby Δ*G*_ATP_ has been fixed at an arbitrary value for both directions of net reaction. For PMF generation linked to ATP hydrolysis, the driving force—in this case Δ*G*_ATP_—has been arbitrarily fixed at −50 kJ mol^−1^; on the other hand, for ATP synthesis at a constant Δ*G*_ATP_ arbitrarily fixed at 50 kJ mol^−1^, the driving force—in this case the PMF—will need to be increasingly greater than the equilibrium PMF of 155.46 mV as thermodynamic efficiency is decreased. As the free energy dissipation is the same for each row of calculated efficiencies in table 1, it is thus a smaller fraction of the driving potential (arbitrarily variable PMF) for ATP synthesis than it is for the driving potential (arbitrarily fixed Δ*G*_ATP_) for ATP hydrolysis, given the numerical method of illustration.

It should be noted that the different rows of results in table 1 are actually simulating the conceivable possibility that there might exist combinations of regulatory phenomena able to regulate an enzyme's activity such that its net reaction rate remains constant in the face of variable intracellular metabolic conditions. In such a case, the net reaction flux (*r*_f_−*r*_b_) would remain constant while the thermodynamic force (Δ*G*_diss_) required to achieve that flux would decrease as the enzyme is activated, leading to a zero-order phenomenological force–flux relationship. There is no conflict between the concept of this simulated scenario and the experimental nonlinear^4^ force-flux relationships published for the F_*o*_F_1_-ATP synthase by Steigmiller *et al*. ([28], fig. 2) and by Petersen *et al*. ([29], figs. 1–3).

Another interesting property of the results shown in table 1 is that all the numerical values except the absolute values of *r*_f_ and *r*_b_ are independent of the arbitrarily chosen net rate of reaction, *r*_f_−*r*_b_. This illustrates that the level of catalysis is capable of determining a wide range of net rates of reaction with no necessary association of high rates with low thermodynamic efficiency. In fact, given the widespread occurrences of such regulatory phenomena for enzyme catalysis as substrate activation, end-product inhibition, cooperativity, storage, mobilisation and—above all these—gene expression, it would come as no surprise if it were to turn out that almost all cellular metabolism runs at high thermodynamic efficiency most of the time. Nonetheless the notion of 100% thermodynamic efficiency is a misconception for the physiological operation of any enzyme that is actually catalysing net chemical or chemiosmotic reaction.

The numerical calculations presented in table 1 illustrate general principles of catalysis that may be gleaned from application of the Rate Isotherm as a kinetic expression of the Second Law. The beauty of thermodynamically determined expressions such as the Probability Isotherm—and its kinetic manifestation, the Rate Isotherm—is that they are entirely aloof from mechanism.

## 10. Conclusion

We have focused on issues pertaining to the stoichiometry and thermodynamic efficiency of chemiosmotic energy transduction by the F_*o*_F_1_-ATP synthase/ATPase. Whereas we have focused primarily on the outstanding experimental work of Toyabe *et al*. [22], our approach is applicable to the equally ingenious experimental accomplishments of other authors referenced in the text. This approach is based on the conviction, arising from the Second Law, that if there is to be a net chemiosmotic reaction then there must necessarily be dissipation of Free Energy. The experimental demonstration of ‘stalling conditions’ is a particular example of thermodynamic reversibility. We attribute free energy dissipation *in vivo* (in the form of heat production) to the internal work required to overcome the resistance of the rotary mechanism and its associated proton channel, as well as to the viscosity of its fluid environment. That is, in the absence of experimentally attached indicators (i.e. actin filaments or gold beads), there is no external *mechanical* work performed by the F_*o*_F_1_-ATP synthase enzyme *in vivo*. Its rotation in either direction, free of external work, is a direct consequence of the electrochemical Free Energy that inheres in the transmembrane proton gradient opposed to the intracellular phosphate potential; the relative probability of rotation in either direction is quantified by the Probability Isotherm or Rate Isotherm.

Nonetheless, the alternative explanation that we have offered to explain the results of Toyabe *et al.* [22] suggests that the internal work of rotary friction in the isolated F_1_-ATPase might be as high as 50% of the free energy available from ATP hydrolysis, and this friction would be the same in both directions of rotation. As new experimental techniques shed more light on the energetic behaviour of the mechanically manipulated F_1_-ATPase [10,11,21], it becomes increasingly important to know whether the friction and torque profiles being measured correspond to those manifest in the intact F_*o*_F_1_-ATP synthase catalysing chemiosmosis. Application of the newer techniques that allow analysis of angular velocity of the *γ*-shaft in the isolated F_1_-ATPase to the study of the intact F_*o*_F_1_-ATP synthase in chemiosmotically functional vesicles would be a significant advance towards clarifying important questions concerning the economy of this enzyme *in vivo* and the overall thermodynamic efficiency of cellular oxidative phosphorylation.

We suggest that the known multiplicity of regulatory mechanisms for the levels of enzyme activity *in vivo* makes it highly unlikely that there is any simple or predictable relationship between net rates of catalysed reaction and thermodynamic efficiency. It may well be that high thermodynamic efficiency is the norm under most physiological conditions. However, 100% thermodynamic efficiency is forbidden by the Second Law whenever a reaction is proceeding with a net rate other than zero.

## Supplementary Material

Force-flux_relations_in_simple_systems_20160106-Revised.xlsx

## Footnotes

1 Chapman *et al*. [23] coined the Probability Isotherm as a corrective measure against widespread misconception and error in the pedagogical treatment of thermodynamics in the bioenergetics sections of most biochemistry textbooks over the preceding 40 years, as historically traced by Larkins *et al.* [24] from the textbook by Lehninger [25] up to the present day.

2 The calculation details of the functions plotted in figures 3 and 4 are given in appendix A.2.

3 The distinction between entropy creation and entropy exchange, and their relationship to thermodynamic efficiency are discussed in appendix A.1.

4 That the nonlinearity of these published force–flux relations is greater than first-order is not to be construed as necessarily indicating force-activation of the enzyme (e.g. by ΔpH) as conceived by Steigmiller *et al.* [28] or Petersen *et al*. [29]. This issue is addressed in appendix A.3 and the Excel file in the electronic supplementary material.

## Data accessibility

The Excel file referred to in Appendix A.3 is available as part of the electronic supplementary material.

## Appendix A

**A.1. Entropy exchange, entropy creation and thermodynamic efficiency**

Consider^5^ a transformation of a system from state *A* to state *B*, represented thus:

A→B.

The First Law of Thermodynamics states that, for such a transformation, the loss in enthalpy (*H*) by the system to its surroundings is equal to the sum of

(i) the heat (*Q*) lost by the system to its surroundings, and
(ii) any work (*W*) performed by the system on its surroundings, thus:
  $$\Delta H_{A\to B}=Q+W.$$

The Second Law of Thermodynamics states that, for such a transformation, the loss in enthalpy (*H*) by the system to its surroundings is equal to the sum of (i) the loss in Gibbs free energy (*G*) by the system to its surroundings, and (ii) the product of the temperature (*T*) and the entropy (*S*) lost by the system to its surroundings by the process of *entropy exchange*, thus:

$$\Delta H_{A\to B}=\Delta G_{A\to B}+T\Delta S_{A\to B}.$$

Equating the First and Second Laws, we have:

$$\Delta G_{A\to B}+T\Delta S_{A\to B}=Q+W.$$

It is a Second Law imperative that the external work performed by the system on its surroundings can never be greater in magnitude than the free energy available from the transformation. Thus,

$$|\Delta G_{A\to B}|\geq |W|.$$

The theoretical limit whereby the work performed is equal in magnitude to the free energy available is known as thermodynamic equilibrium; at this condition no transformation takes place, i.e., no work is done and nothing happens. In order for any transformation to occur, a non-zero amount of free energy must be dissipated as heat. The thermodynamic efficiency (*η*) of transformation is the work (*W*) performed, expressed as a fraction of the free energy available, thus:

$$\eta =W/\Delta G_{A\to B}\leq 1.$$

For all non-equilibrium situations, the free energy is either conserved as work or dissipated as heat in the process of *entropy creation*, thus:

$$\Delta G_{A\to B}=\eta \Delta G_{A\to B}+(1-\eta )\Delta G_{A\to B}=W-\Delta G_{\mathrm{diss}},$$

where Δ*G*_diss_=−(1−*η*)Δ*G*_*A*→*B*_. Thus, the total heat (*Q*) lost by the system to its surroundings derives from two entropic sources:

(i) the entropy (Δ*S*_*A*→*B*_) *exchanged* between the system and its surroundings, inescapably bound to the material transformation *A*→*B*; and
(ii) the entropy created within the system and its surroundings through the free energy dissipation required by the Second Law, thus:
  $$\Delta S_{\mathrm{created}}=\Delta G_{\mathrm{diss}}/T$$
  and depending on the thermodynamic efficiency at which the transformation takes place.

**A.2. Calculation details for the plotted curves of figures 3 and 4**

Figure 3 shows simulated plots of the respective fractions of observed 120° steps in the forward and reverse directions as functions of the applied torque over the domain shown at intervals of 1 pN nm rad^−1^ of torque. These observed steps are assumed to comprise a torque-dependent, thermodynamically determined catalytic component, *r*_f_ and *r*_b_ in each respective forward and backward direction, and a torque-independent level of contamination, *c*=*c*_f_=*c*_b_ in each respective direction. The level of contamination, c, was explored over a stepped domain from zero (no contamination) to 100% (parity with the maximum unidirectional level of true catalytic stepping in the stalling condition, assumed to be 17.5 s^−1^).

The calculation algorithm was coded as follows (code to the left and comments to the right of the emdashes):

**Table:**

W = N*1e-21*120/Radian	—	W = work; N = number of pN nm rad^−1^ of torque; 120 = degrees of the catalytic step;
		Radian = the number of degrees in a radian.
DeltaMudiss = −DeltaMuATP − W	—	Δ*μ*_diss_=−Δ*μ*_ATP_−*W*=65.2e-21 pN nm − Work.
rateRatio = exp(DeltaMudiss/(kB*T))	—	Rate ratio = exp(Δ*μ*_diss_/(k_b_T);
		k_b_= Boltzmann constant = 1.3806488e-23 J K^−1^.
rb = Vmax/(1 + rateRatio)	—	$r_{b}=V_{\max}/(1+\exp(\Delta \mu_{\mathrm{diss}}/(k_{B}T))$; $V_{\max}=35\;s^{-1}$.
rf = rateRatio*rb	—	$r_{f}=\exp(\Delta \mu_{\mathrm{diss}}/(k_{B}T))r_{b}$
obsb = rb + contamination	—	Observed backward stepping rate = true catalytic backward stepping rate + contamination;
		contamination is identical in both directions and preset at levels ranging from zero to 17.5 s^−1^.
obsf = rf + contamination	—	Observed forward stepping rate = true catalytic forward stepping rate + contamination.
pobsf = obsf/(obsf + obsb)	—	Probability of observing steps in the forward direction as a fraction of the total forward and backward stepping rates observed. This calculated probability is the quantity plotted in the curves sloping *up* from left to right (synthetic direction) in figure 3.
pobsb = obsb/(obsf + obsb)	—	Probability of observing steps in the backward direction as a fraction of the total forward and backward stepping rates observed. This calculated probability is the quantity plotted in the curves sloping *down* from left to right (hydrolytic direction) in figure 3.
Two further lines of code were used to calculate the curves plotted in figure 4, thus:		
DeltaMudiss = kB*T*ln(rateRatio)	—	Δ*μ*_diss_=k_b_T ln(r_f_/r_b_) This calculation is actually redundant, given the second line of code shown above.
		It shows the true Probability Isotherm for the uncontaminated catalytic steps.
DeltaMuobs = kB*T*ln(obsf/obsb)	—	Δ*μ*_obs_=k_b_T ln(observed forward stepping rate/observed backward stepping rate). This shows the apparent Probability Isotherm as contaminated with catalytically fruitless steps

**A.3. Nonlinear force-flux relations in simple systems**

An Excel file has been provided as part of the electronic supplementary material, allowing user interaction to explore the nonlinear force-flux relationships that obtain for (i) simple first-order diffusion and (ii) the simple second-order chemical reaction, 2*A*→*B*. It is suggested that users experiment with different values for the GREEN cells in the two respective spreadsheets.
